# Domperidone‐induced pityriasis rosea‐like drug eruption

**DOI:** 10.1002/ccr3.5674

**Published:** 2022-04-05

**Authors:** Sarra Saad, Rima Gammoudi, Nihed Abdessayed, Mohamed Denguezli

**Affiliations:** ^1^ Department of Dermatology Farhat Hached university hospital Sousse Tunisia; ^2^ Department of Anatomopathology Farhat Hached university hospital Sousse Tunisia

**Keywords:** pityriasis rosea‐like domperidone drug eruption, pregnancy

## Abstract

Pityriasis rosea is a common, acute, self limiting inflammatory skin disease. Pityriasis rosea‐like eruptions (PR‐LE) have been reported after drugs. The clinical presentation of PR‐LE can be distinguished from pityriasis rosea. We reporte a 41‐year‐old woman who developed PR‐LE 5 days after administration domperidone.

## Abstract 2

The clinical presentation of Pityriasis rosea‐like eruptions (PR‐LE) can be distinguished from pityriasis rosea. Herald patch and prodromal systemic symptoms are absent in PR‐LE, and lesions are more confluent forming large itchy lesions.

## 1 CASE OBSERVATION

**FIGURE 1:**
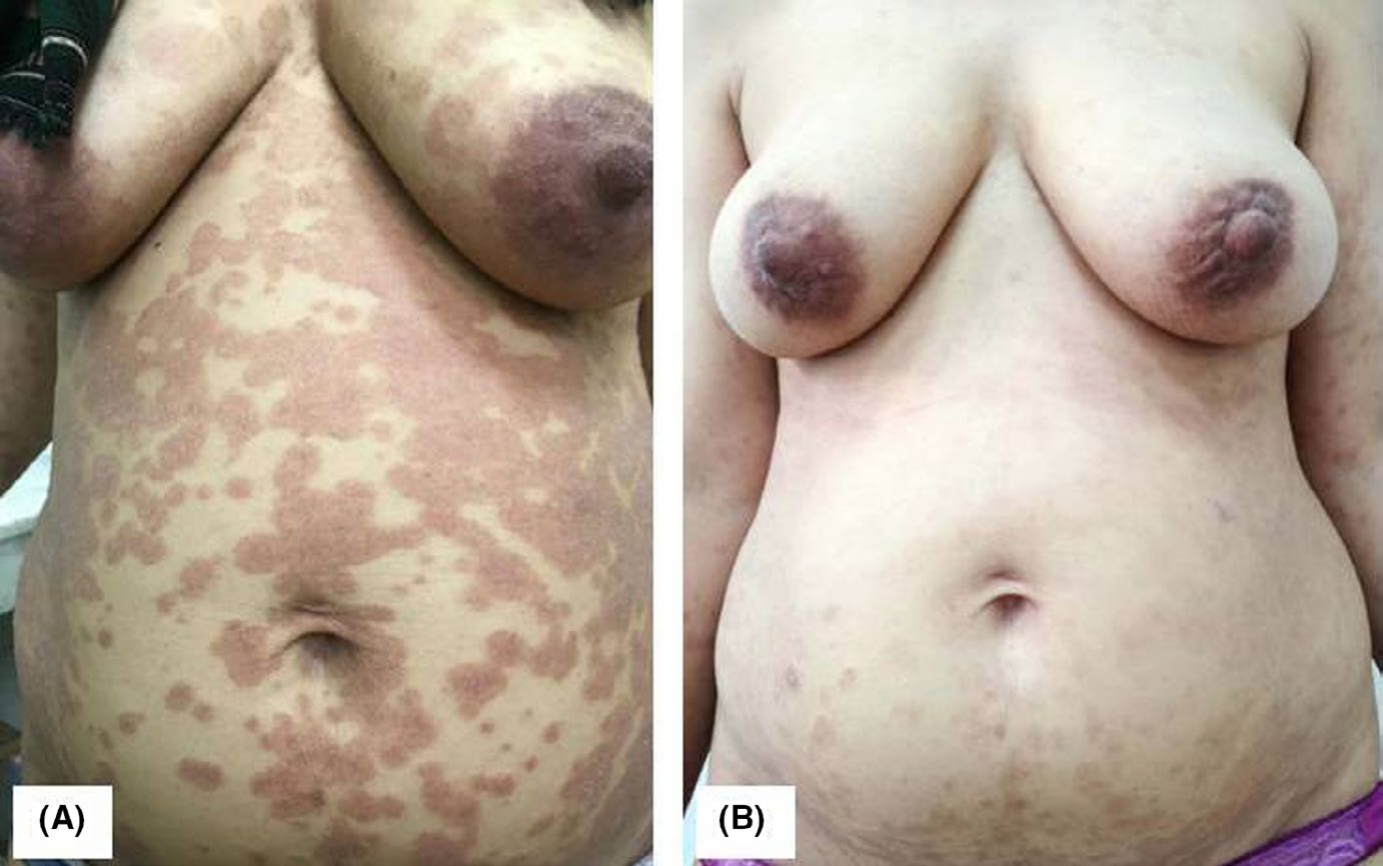
(A) Erythematous and squamous lesions on the trunk. (B) Without topical treatment, the lesions disappeared two weeks after discontinuing domperione

**FIGURE 2:**
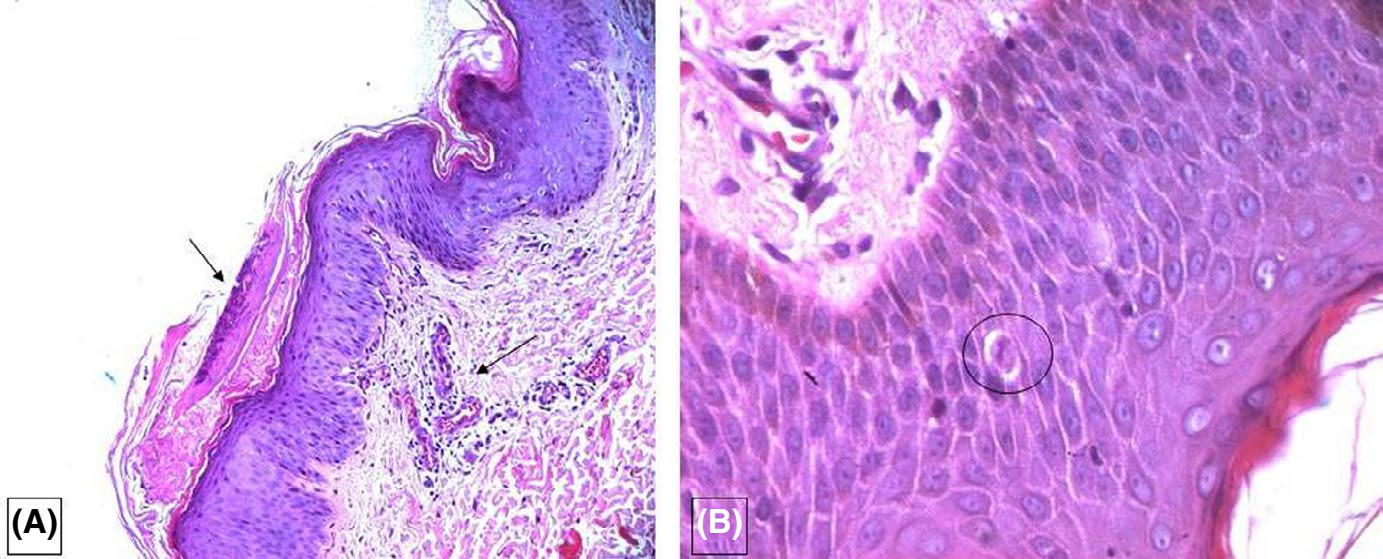
(A) (HE ×100), Parakeratotic hyperkeratosis in front of acanthotic rete pegs. Perivascular lymphocytic and eosinophilic infiltrate in dermis. (B) (HE ×200): Rare apoptotic bodies in the epidermis

A 41‐year‐old woman, G2P1 presented at the 12th week of pregnancy with a pruritic eruption on the upper and lower limbs, neck, and trunk. The eruption appeared 5 days after the administration of domperidone. No symptoms of viral infection were observed in the previous week. The dermatological examination revealed scattered erythematous and squamous lesions on the arms, chest, and abdomen (Figure 1A), and confluently scaly plaques of the lower back. Histological examination revealed signs of a drug reaction (Figure 2). The rash disappeared 2 weeks after discontinuing the treatment (Figure 1B).

## 2 DISCUSSION

Pityriasis rosea is a common, acute, self‐limiting inflammatory skin disease due to the endogenous systemic reactivation of human herpesvirus‐6 (HHV‐6) and/or ‐7 (HHV‐7). Pityriasis rosea‐like eruptions (PR‐LE) have been reported after drugs.^1^ Recently, several cases have been reported of PR‐LE after COVID‐19 vaccines.^2^ The clinical presentation of PR‐LE can be distinguished from PR. Herald patch and Prodromal systemic symptoms are absent in PR‐LE and lesions are more confluent forming large itchy lesions.^1^

Skin reactions to domperidone have been described in two cases represented by systemic lupus‐like syndrome and general diffuse erythema with some pustules. The pathophysiology between domperidone and these different reactions is not well understood, it is probably multifactorial and complex.

## CONFLICTS OF INTEREST

None.

## AUTHOR CONTRIBUTIONS

Dr Sarra Saad involved in writing the manuscript and submitting the revised article. Dr Rima Gammoudi out the analysis of the manuscript. Dr Nihed Abdessayed involved in writing the histological part of the manuscript. Dr Denguezli Mohamed supervised and approved the revised manuscript.

## ETHICAL APPROVAL

None.

## CONSENT

Written informed consent was obtained from the patient to publish this report in accordance with the journal's patient consent policy.

## Data Availability

None.

## References

[1] Drago F , Ciccarese G , Parodi A . Pityriasis rosea and pityriasis rosea‐like eruptions: how to distinguish them? JAAD Case Rep. 2018;4:800‐801. 30246131 10.1016/j.jdcr.2018.04.002 PMC6142012

[2] Carballido Vázquez AM , Morgado B . Pityriasis rosea‐like eruption after Pfizer–BioNTech COVID‐19 vaccination. Br J Dermatol. 2021;26. doi:10.1111/bjd.20143 PMC8239510 33904157

